# Using Readable English leads to reading gains for rural elementary students: An experimental study

**DOI:** 10.1371/journal.pone.0288292

**Published:** 2023-07-20

**Authors:** Joanne Veatch Coggins

**Affiliations:** 1 University Studies, Middle Tennessee State University, Murfreesboro, Tennessee, United States of America; 2 Department of Reading Research, Righting Reading, Wartrace, Tennessee, United States of America; Tallinn University: Tallinna Ulikool, ESTONIA

## Abstract

**Objectives:**

This study measured whether and to what degree Readable English effectively improves reading fluency and comprehension skills of adolescent learners.

**Methods:**

This experimental study (*N* = 855) measured the efficacy of two different multiple component reading programs for students in grades three, four, and five. Students were pre-and post-tested using EasyCBM Grade Level Reading Benchmarks. Students scoring at/below the 30^th^ percentile on either benchmark were also assessed with the WRMT-3 Passage Reading Comprehension and Oral Reading Fluency measures. Students received 45–60 instructional hours in either Readable English or Amplify CKLA during their regular ELA class.

**Results:**

Students who received Readable English instruction significantly outperformed students in the typical practice condition on all measures of reading fluency and comprehension. The intervention condition’s EasyCBM benchmark reading rate (*m* = 29.6 WCPM), reading accuracy (*m* = 3.1%), and comprehension (*m* = 1.9) surpassed the control group’s reading rate (*m* = 17.4 WCPM), accuracy (*m* = 0.7%), and comprehension (*m* = 0.7) WRMT-3 ORF showed students in the Readable English intervention condition (*m* = 13.4 growth scale values [GSV] and *m* = 1.0 grade equivalency) outscored students in the control condition (*m* = 7.8 GSV and *m* = 0.5 grade equivalency). WRMT-3 Passage Comprehension showed Readable English students (*m* = 11.0 GSV and *m* = 0.9 grade equivalency) outgrew control condition students (*m* = 4.4 GSV and *m* = 0.3 grade equivalency).

**Conclusions:**

In a school year fraught with pandemic instructional interruptions and learning loss, students in grades 3–5 who received Readable English instruction closed reading gaps. The meaningful gains in reading rate and accuracy experienced by students in the intervention group will give exponential word reading volume dividends to students able to read text faster and more accurately going forward, helping them become more skilled readers.

## Introduction

According to the 2022 National Assessment of Educational Progress only 33% of fourth graders and 31% of eighth graders are proficient readers, with significant racial disparities apparent between white students and all other races [1]. Clearly many students leave high school without the reading ability needed to fully access all the opportunities available to skilled readers. Educational tracks for the reading ‘Haves’ and ‘Have Nots’ may diverge in many places, but there are two main places students get derailed from the path to reading proficiency, fluency and understanding. The purpose of this article is to review basic reading development to understand where reading becomes difficult for children, identify specific reading processes integral to reading, clarify what is needed to support good reading development, and consider the results schools achieved from implementing Readable English in grades three through five.

The trajectory for non-proficient adult readers begins in elementary school with low or even low-average reading fluency [2]. Every year that reading skills deficits of young readers are not remediated compounds reading comprehension problems adult readers will face [3, 4]. Teachers beyond third grade generally do not have the time or instructional skills needed to remediate students’ reading fluency skills, so students with reading skills deficits often are passed along the grades [5, 6]. A basic knowledge of reading skills development allows us to predict that the older students are, the greater the impact reading fluency deficits have on reading comprehension [7, 8]. As reading for information increasingly becomes the focus of instruction, students with weak reading skills suffer in a myriad of ways that contribute to an education debt that can transcend generations [9].

The social effects of low reading ability influence virtually every aspect of a person’s life, impacting financial, social, and educational well-being, as well as physical and mental health [10, 11]. No one likes to feel inadequate or to appear incompetent, and this is especially true of children. Students understand at an early age that reading is a key part of their learning responsibility [12]. As nonproficient readers age, they develop coping mechanisms to cover their reading difficulty that varies in severity and range from guessing or skipping the words they cannot read to disruptive behaviors that divert from engagement in reading activities [8]. Other students who struggle with reading seem to disappear in the classroom to avoid drawing notice and are absent frequently. Stories abound of children, adolescents, and adults feigning hearing loss and poor vision rather than admit they cannot read [12]. When reading is an integral part of academic activities, not being a good reader can be hard on a student’s self-esteem and emerging sense of self and can be a profound source of shame through no fault of the child [13].

Early literacy development begins before students enter school and is influenced by many factors, including the amount of verbal language they are exposed to, the reading habits of their parents, and whether they were read to as very young children [14, 15]. Before and during kindergarten, students develop awareness of the individual sounds that make up words. Automatic recognition of word sounds (phonological awareness) and the ability to manipulate basic letter sounds (phonemic awareness) are integral in learning to read [10]. Students usually begin sounding out two and three letter real words and word parts during kindergarten [10].

Through third grade, reading fluency is the primary focus of reading instruction, with the focus shifting from reading individual words to reading increasingly complex sentences and connected text [16]. The number of words students read correctly and accurately continues to increase dramatically through third grade. Around fourth grade the focus on reading instruction shifts from reading fluency (learning-to-read) to reading comprehension (reading-to-learn), though reading fluency also continues to increase [17]. Beginning in fourth grade students read increasingly complex text to learn new concepts and develop an expansive vocabulary of rare and academic words [18]. This read-to-learn shift is a sharp dividing line between students with adequate reading fluency skills and those who have fluency skills deficits [2, 19]. Educators refer to this as the “fourth-grade slump” as student scores on end-of-year state tests suddenly dip or plateau.

Students with unexpected poor comprehension begin to emerge in fourth grade, as students with average or below average reading fluency struggle to understand what they are reading [20, 21]. Students who appeared to be on track academically with low-average or average fluency through grade three begin encountering longer sentences with academic and multisyllable words that require increasing cognitive resources for comprehension [22]. Students spending finite cognitive resources on reading the words on the page may struggle to comprehend new content or struggle to assimilate rapidly expanding vocabulary and knowledge bases [18]. Unable to develop the requisite vocabulary or build foundational knowledge (schema), new learning is significantly reduced through a combination of non-comprehension, misunderstanding information, or insufficient schema (background knowledge) on which to attach new knowledge [16, 22, 23].

As a result, students in fourth grade who lag slightly behind their peers and who may have seemingly small reading skills deficits are slowly left further behind during grades four and five [21]. Beyond grade six those seemingly small reading skills discrepancies are amplified in students who have not built the core knowledge and have not developed the academic vocabulary their peers with strong reading skills have acquired [16, 24]. Disfluent readers, unable to read the increasingly complex text of core curriculum, do not develop the rich vocabulary and schema of proficient readers [24]. Every year struggling readers get left further behind their peers as the reading skills gap widens [5, 25].

### The mechanics of reading: A lot of moving parts

Successful literacy development can be visualized as a tunnel spiderweb: reading skills build and spiral, integrating multiple mutually supportive processes as new readers progress through the stages of reading. Relying on verbal vocabulary to recognize words, beginning readers decode letters from blended sounds into words to read them aloud [10]. Individual words are decoded and quickly and accurately recognized. Rereading words several times adds them to the reader’s mental lexicon (word memory) where they can then be read as whole words, and word reading becomes increasingly automatic as known words are recognized on sight [26]. This process of orthographic mapping is how we create mental images of words.

As word reading becomes easier and faster, reading practice and the volume of words read increases; and typically developing beginning readers progress from reading individual words to reading connected text [26]. Exposed to increasingly complex text, developing readers continue to encounter new words which must be decoded and whose meanings must either be recognized or inferred [27, 28]. Word level reading skills strengthen, freeing additional cognitive resources to make meaning from text [24].

Students vary in the ways they form connections between word identification and lexical memory, so a variety of scaffolds are needed to support reading development [29]. The *lexical quality* of words readers add to their mental lexicon varies in degree of accuracy and depth of knowledge of the words, and is measured in terms of accuracy of spelling, pronunciation, meaning, and usage [30]. Readers with higher lexical quality representations of words understand meanings of words used in differing contexts and can use those words. Higher lexical quality of words means faster and more accurate word recognition. When word recognition is automatic, readers can focus on creating meaning from the text rather than on reading the individual words, increasing comprehension [7]. When these processes work smoothly novel words are quickly assimilated, vocabulary grows, and text meaning is synthesized and added to existing schema [28, 29]. In addition to the complex processes involved in reading, the English language offers particular reading challenges.

English has adopted words from hundreds of languages spoken around the world, resulting in an extremely large and very rich vocabulary [31]. The spellings and pronunciations of adopted words largely have been maintained, meaning many words do not follow basic phonics rules. This creates a tension between understanding the morphology or the phonology of a word. The morphology of an unknown word is seen in its spelling and gives evidence for potential meaning (e.g., *foursome*), but just as frequently a word’s pronunciation is a clue to its meaning (e.g., *wind*) [18, 32]. Adopting words from other languages with grapho-phonemic correspondences that differ significantly from English often means that words are not pronounced the way they are spelled (e.g., *could*). Weak grapho-phonemic correspondences mean that many words are not decodable and must be memorized as whole units. Often words change pronunciation with tense changes (e.g., *read*) or when affixes are added (e.g., *live*, *alive*, and *livable*) and they are pronounced differently among various English-speaking populations.

Given the orthographic and phonemic challenges learning to read and write in English poses, it is unsurprising that most early grades teachers have negative perceptions of scripted phonics programs [33]. Campbell found that early grades teachers are averse to using explicit and systematic phonics programs because they do not like the rigidity of scripted instruction or teaching skills in isolation. Despite current interest in the Science of Reading, teachers continue to perceive explicit reading skills instruction as “drill and kill,” and the widespread belief that rich exposure to reading causes children naturally to grow into skilled readers persists [34]. The dichotomy between a theoretical conception that explicit reading skills instruction is the best way to create skilled readers and actual classroom practices is largely due to time constraints and an inability to embed newly learned reading skills across the curriculum [33, 34]. In other words, reading instruction happens during the reading class time and other subject areas are equally compartmentalized. Teachers continue to report that it is difficult to conceptualize and integrate reading instruction with math, social studies, science, and other core content areas [33–35]. An inherently complex task, students need thousands of hours of practice to become proficient readers [3], necessitating cross-curricular reading instruction to get both enough reading instruction and practice reading.

The continuum from decoding to reading fluency to reading comprehension is not always a smooth path [21]. Though fundamental to learning, reading is not easy for most students, and a breakdown anywhere in the cycle negatively impacts reading comprehension [2]. Many discrete skills work multi-directionally to support good reading, which means that having a skills deficit in one or more areas can cause reading to be difficult. The greater the degree of reading difficulty a student experiences, the greater the likelihood that multiple reading skills processes need to be supported [8]. The potential number of instructional targets and widespread lack of reading proficiency necessitate the use of robust reading programs that facilitate growth of interrelated skills in both foundational reading fluency and reading comprehension [23, 36].

Ideally, reading programs should be built upon an asset-based approach to reading instruction that incorporates the individual interests and strengths of students [9]. Readable English is able to provide this type of individualized instructional programming, while incorporating multilevel fluency and comprehension activities for students with a wide array of reading skills strengths and weaknesses. Anecdotal teacher, parent, and student reports indicate Readable English substantially helps students grow into much stronger readers, but this is a new reading program. A pilot study [37] found that the program was effective for high school students with reading disabilities. Middle school students (grades 6–8) with average and below-average reading skills who received Readable English instruction made significant gains in reading fluency and comprehension during the pandemic [38]. However, there are no peer-reviewed studies of the effectiveness of Readable English instruction with elementary school students. This study provides important research into whether and how effective Readable English, a program rapidly gaining popularity in many parts of the United States, may be for older elementary-age students.

### The present study

The purpose of this study was to ascertain whether and to what degree Readable English effectively improves reading fluency and comprehension skills of students in grades three, four, and five. Evaluation goals of this sustainable, multiple component reading program included these key criteria:

- Is easy for teachers to implement with fidelity.
- Includes multiple components for teaching foundational reading skills (i.e., phonemic awareness, phonological awareness, phonics, orthographic mapping, morphological awareness, syntax) that support reading fluency, vocabulary building, comprehension, and writing.
- Includes spiral instruction to identify and remediate skills deficits as they occur.
- Embeds literacy across the core curriculum to maximize both time spent reading and exploring new subject matter.

Readable English is a multiple component reading program that helps teachers embed literacy throughout the curriculum. Other teaching methods require memorization of dozens of rules that are not widely applicable to even the most common English words. Words that do not follow the rules have to be memorized and recognized on sight. Using Readable English all words follow phonetic rules using glyphs to stabilize pronunciation and decoding, and silent letters are greyed out. Longer words have pronunciation breaks. Rather than providing leveled readers, this program makes all text completely decodable–including grade level curriculum.

Multiple component reading programs include many activities in each of three main domains: reading fluency, vocabulary building, and reading comprehension. Strong asset-based reading interventions focus on scaffolding reading at the students’ instructional level in the context of reading, math, and written language, and provide multiple types of activities that reinforce individual reading skills [7]. An example of one type of multiple component reading activity could include the teacher identifying unknown vocabulary and prereading words to connect new vocabulary with prior learning, completing a graphic organizer to synthesize thoughts and organize writing, and working on several short reading fluency and spelling activities all organized around a common theme or central text.

Findings from prior studies indicate that multiple component interventions are particularly effective at remediating reading fluency or reading comprehension skills for students in elementary or middle school grades [36–38]. However, interventions that address fluency as a multilevel construct supporting multiple processes tied to reading comprehension rarely extend beyond repeated reading of text [7, 8]. Even fewer studies have investigated the effect of multiple component reading programs on students’ reading fluency *and* text comprehension across elementary grades [8]. No large-scale studies have previously examined the effectiveness of Readable English across a continuum of reading ability levels in grades three through five.

Because students begin moving away from learning to read in third grade toward reading to learn new information, students with average or lower reading skills need ongoing multiple component supports to catch up to and keep up with their peers [2]. Third, fourth, and fifth grade students whose education was disrupted during the prior school year particularly needed robust instruction to improve their reading. This study investigated whether and to what degree Readable English helped elementary students with average to low reading fluency and reading comprehension improve those reading skills compared with the districts’ typical reading instruction.

In the United States, individual state departments of education have the authority to determine what educational programming and instruction is appropriate. “Local control” means each state either creates a vetted list of appropriate reading programs and/or interventions or they recommend school districts use research proven or research-based reading programs. The downside of this freedom is that nationwide use of instructional “best practices” has not elevated American students’ overall reading abilities. In fact, in 2022 American students recorded the largest across the board decline in reading scores in more than thirty years (National Center for Educational Statistics). When “best practices” fail, teachers must find “better” practices, including innovative teaching programs and strategies. The freedom to try innovative new reading programs such as Readable English is the upside of states’ local control of education because it provides an opportunity to strive for and measure academic growth in a real-world classroom setting. This study measured the efficacy of such an innovative reading program during the 2020–2021 school year when students worldwide experienced significant, lasting learning loss due to the pandemic.

Two main points of inquiry guided this research. When compared with other students in grades three, four, and five receiving typical practice instruction using Amplify Core Knowledge Language Arts (CKLA) multiple component reading program:

1. Does Readable English instruction promote superior growth of reading fluency? Do oral reading fluency, reading rate measured by WCPM, and/or reading accuracy significantly improve for students in the intervention condition?
2. Does Readable English, a program supporting fluency at multiple levels, meaningfully improve reading comprehension of students in the intervention condition?

## Materials and methods

### Procedures

Study proposal and protocols were reviewed and approved by Integ Review IRB (protocol number 1228). Pre-test assessments were administered during the schools’ usual benchmark testing windows in mid-September to mid-October 2020 and post-testing occurred from mid-April to mid-May 2021. Individually administered tests were conducted by trained administrators, teachers, or school psychologists under the direct supervision of the research team’s psychometrician. The intervention program occurred from mid-October 2020 to mid-April 2021 (i.e., between pre- and post-testing). Eighty intervention hours were planned, but due to rolling COVID school closures and weather events students received forty-five to sixty instructional hours (*M* = 56 hours).

### Research design

This research study (*N = 855)* was a multisite, experimental design blocked by grade that encompassed fifty-five teachers at ten rural schools located in three rural districts in both Indiana and Tennessee. Three schools were assigned to the typical practice condition, four schools were assigned to the intervention condition, and three schools had grade levels of students assigned to both condition groups. At schools participating in both typical practice and intervention conditions, three teachers who taught multiple grade levels taught students in both study conditions. Average annual enrollment for the ten participating schools was 1,400 students, with between 49.3% and 59.5% of students qualifying for free or reduced-price lunch [39]. Students with limited English proficiency represented less than 2% of the total student populations of participating schools and were excluded from the study. Grade levels of Reading/English Language Arts classes within each school were assigned to either the intervention or typical practice condition. Special Education teachers followed grade level research design blocking to provide either Readable English instruction to their students in the intervention condition or they continued with the usual instruction for students assigned to the typical practice condition. *A priori* power analyses indicated that sample sizes per grade level treatment condition needed to include at least 105 participants to be sensitive enough to find moderate effect sizes with significance of α = 0.05. These sample size goals were met, and student demographics are described in Table 1.

**Table 1:** Descriptive student demographics by intervention condition.

Variable	Readable English (*N* = 441)		Typical Practice (*N* = 414)	
	*n*	%	*n*	%
Gender				
Female	222	50.3	196	47.3
Male	219	49.7	218	52.7
Ethnicity				
Asian	2	0.5	0	0
Hispanic/Latino	57	12.9	62	15.0
Black/African American	8	1.8	13	3.1
White	374	84.8	339	81.9
Identified for Special Education	98	22.2	83	20.0

Chi-square tests of categorical variables (e.g., gender, ethnicity) showed no significant differences in student characteristics between the treatment and typical practice group. Independent sample *t* tests were conducted to evaluate group equivalence for pretest measures. Analyses indicated there were no significant differences between the treatment and typical practice groups on pre-test WRMT-3 measures. However, there were statistically significant differences between the groups on all three EasyCBM pre-test measures and these were controlled for during data analysis.

### Attrition analysis

Of the 1023 students who began the study, 855 (83.6%) completed both pre- and post-test assessments. Comparisons of demographic and pre-test scores of the 168 attritors in the control and treatment conditions showed no significant differences in any variable between the students who left the study and those who completed post-testing. There was no significant difference between the number of students who left the study in the control (n = 87) and treatment (n = 81) conditions. Students were excluded if they transitioned from in-person to online instruction or were not in attendance to complete either pre- or posttesting.

### Intervention training and supervision

Teachers received initial and ongoing instruction in how to teach the Readable English program, and teachers were encouraged to use the conversion tool to convert a variety of core curriculum content from standard English into text with the Readable English mark-up. At schools using Readable English, both the general and special education teachers used Readable English as their regular Reading or English Language Arts (ELA) instruction. At the beginning of the school year all teachers received two full days of intervention implementation training, and teachers assigned to Readable English intervention condition groups were provided with an intervention coach to monitor instructional fidelity and to assure proper program pacing. Coaches provided teachers with weekly lesson plans and teaching guide manuals that included scope and sequence for each phase of the intervention. Both condition groups introduced main lesson ideas in whole group settings, before students received direct instruction in small groups with the teacher. During small group instruction, students worked in group workstations when not working with the teacher.

### Intervention fidelity

To monitor instructional fidelity coaches and teachers in the Readable English intervention condition group checked in either virtually or in-person every week school was in session (i.e., twelve to sixteen times). Coaches modeled lessons and observed teacher instruction with students, later providing feedback and encouragement. Teachers were encouraged to use the conversion tool for cross curricular reading assignments and were provided multiple demonstrations of both how to use the conversion tool and practical exemplars. Coaches monitored teacher progress through the Readable English intervention for appropriate pacing, and to be certain teachers were using all relevant intervention materials and practices. Teachers in the typical practice control group received support from districts’ academic coaches to assure district scope and sequence guidelines for Amplify CKLA were followed.

### Description of Readable English intervention condition

**Fig 1:**
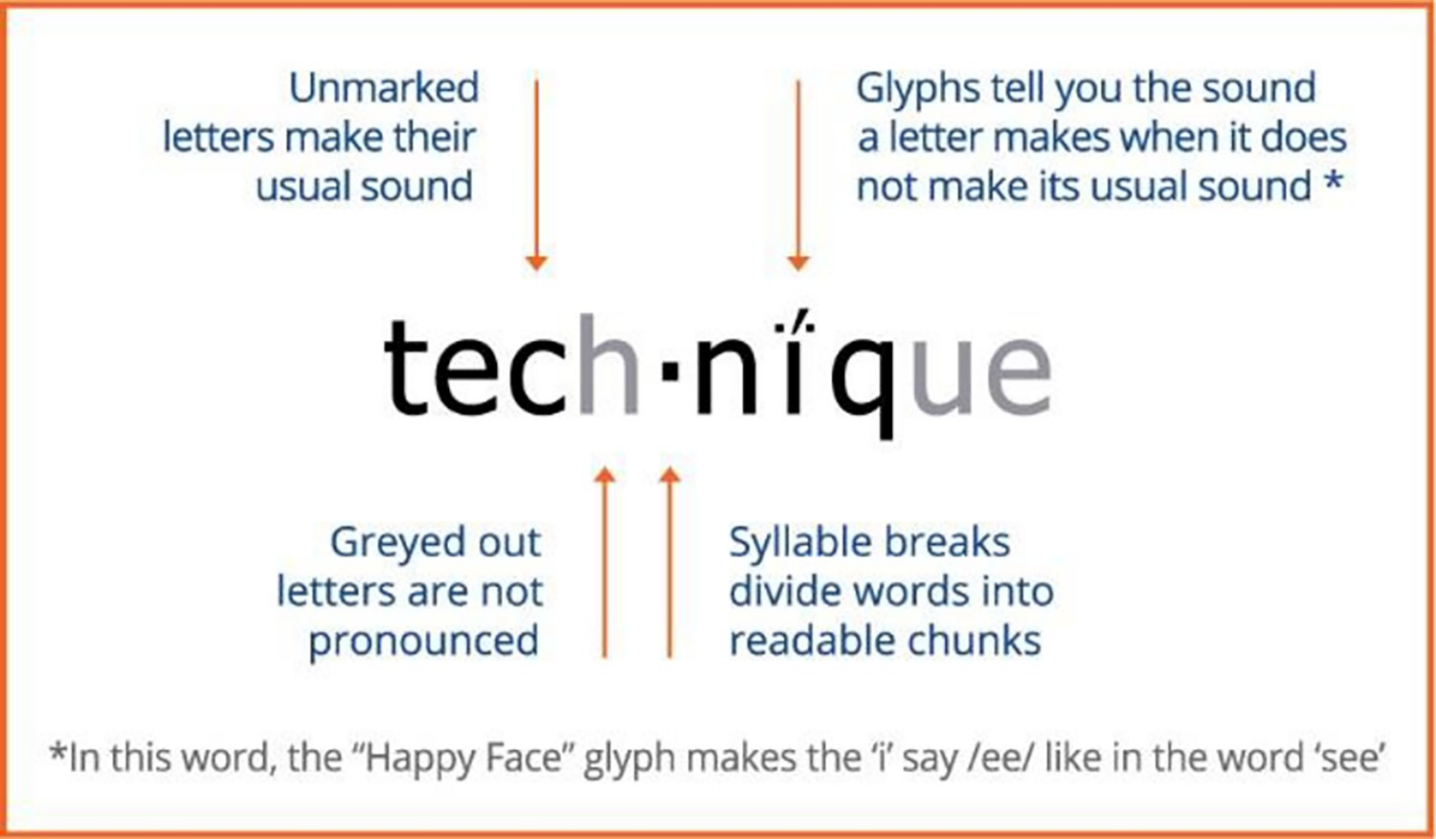
Example word with Readable English mark-up. Readable English© (2023). Reprinted with permission [40].

Readable English uniquely targets reading fluency skills deficits and provides individualized reading scaffolding without significant teacher involvement. Rigorous word and nonword decoding skills practice tasks and games are integrated into the computer-based program that guides the students’ individual learning paths. Students practice reading increasingly complex levels of text (i.e., connected text, passages, narratives, and nonfiction works), with integrated checks for student reading comprehension. Various grade level text is available online in the student eReader, or teachers or students can choose any other text they want to read. The Readable English mark-up makes words completely phonetic in three ways. First, letters that do not follow their primary standard English pronunciation are marked with a glyph like a diacritical mark that cues the student to the letter’s accurate pronunciation. Second, letters that do not make a sound are visible but are grayed out to indicate their silence; and third, pronunciation breaks are indicated by a dot to support reading of multisyllable words (see Fig 1).

During the six weeks of Phase One of Readable English, students learned the twenty-one glyphs through songs, rhymes, movement, and games. Phase Two (which ranged from six to twelve weeks) focused on decoding, spelling, and fluency skills using interactive worksheets, games, and text through teacher led small group and student independent workstations. All activities were performed either on the computer or printed out by the teacher and used independently of the computer. During Phase Two students began using the Readable English conversion tool to read core curriculum. Teachers were encouraged to convert core content from across the curriculum, including books, worksheets, and project instructions, into the Readable English mark-up. Phase Three (ten plus weeks) built on the fluency skills learned in Phases One and Two and focused on reading comprehension, writing skills, and vocabulary building.

#### The conversion tool

The Readable English mark-up makes text transparently decodable. This eliminates the need for students to guess at word pronunciation, though it does not eliminate the guessing habit many students develop as a compensatory reading strategy. The conversion tool can convert text that has been cut and pasted from the computer screen, as well as a wide variety of file types (e.g., Word documents, PDF text, PDF image, JPG, PNG, TIFF, TXT, HTML), so most types of text can be converted to the Readable English mark-up. Teachers can convert materials and add them to the students’ individual eReader libraries, along with vocabulary word lists. Students can add and delete words to their personal vocabulary list (S2 File). These functions make it possible to scaffold cross-curricular content to students’ independent reading levels and incorporate vocabulary from math, science, and social studies with ELA material.

The conversion tool gives students autonomy to individualize their own reading materials. They can choose what text to convert and either print it or read it online. E-reader setting options include font size, word spacing, letter spacing, line spacing, font color and background color of text. Students choose whether they want to view silent letters greyed out. The Readable English mark-up can be turned off for the entire text or for individual words the student has learned to read. Right clicking on a word turns the Readable English mark-up on or off. Left clicking on a word pulls up a definition, and options to hear the word read, add it to a vocabulary list, or translate the word into one of forty other languages. The student can bookmark the last page read.

Using the conversion tool, students choose what to read and how they want to read it, and teachers can source materials from their favorite projects, textbooks, websites, programs, etc. Teachers can integrate cross-curricular content and provide a high level of individualized student support many children need to read grade level curriculum to build academic vocabulary and expand knowledge bases. The Readable English curriculum targets multiple reading skills components and multidirectionally supports all currently defined reading processes, providing important skills practice for students with a range of reading skills deficits.

### Description of typical practice Amplify CKLA condition

Teachers used Amplify CKLA and followed district grade level scope and sequences. This program offers foundation reading skills assessments (DIBELS 8) in a student dashboard so teachers can monitor student reading progress. Reading passages, worksheets, and skills games are available for online learning. Amplify CKLA had been used by all the study schools for a minimum of three years and includes activities designed to teach, explicitly and systematically, multiple literacy skills involved in reading fluency and comprehension. Students in both condition groups received equal instructional time.

### Teacher test administration training

Teachers in both the treatment and control conditions received training in how to properly administer EasyCBM reading benchmarks. All teachers were reminded of benchmark testing windows and individually advised on missing student data during each testing window. To ameliorate potential Hawthorne effects, all teachers in both conditions were provided student benchmark score reports. All teachers and administrators also had equal access to the EasyCBM student scores portal.

### Measures

#### EasyCBM reading benchmarks

The EasyCBM Common Core State Standards (CCSS) Basic Reading benchmark assessment [41] is a twenty-five-item group administered online test measuring comprehension skills of nonfiction and informational text. A sixty-second individually administered test, Passage Reading Fluency [42] measures reading rate WCPM and reading accuracy. Using these two metrics a reading fluency percentile is calculated. The benchmark tests are grade-based, normed, and delivered during the Fall, Winter, and Spring testing windows. Internal consistency for the CCSS Basic Reading test is high, with a median Cronbach’s alpha of 0.87 and median split-half reliability of 0.76 and 0.83 across all measures [43, 44]. Researchers chose the EasyCBM for its ease of use across multiple grades and the analytics available to teachers and parents. All students in all schools were assessed with the EasyCBM benchmarks. The Fall benchmark was used as a universal screener to determine which students might have significant reading skills deficits.

Because students reading well-below grade level may make reading skills gains that are not reflected in tests beyond their independent reading level, additional and more sensitive assessments were used. Students scoring at or below the 30^th^ percentile on either the EasyCBM Passage Reading Fluency or CCSS Basic Reading test were further assessed using the Woodcock Reading Mastery Tests, 3^rd^ Ed. (WRMT-3) Passage Comprehension and Oral Reading Fluency subtests [45].

#### WRMT-3

The WRMT-3 is a battery of individually administered assessments measuring reading achievement skills of students in pre-kindergarten to grade twelve. Tests have grade-specific start points and items are increasingly difficult until the discontinue rule is met. Study participants were assessed with the WRMT-3 Oral Reading Fluency and Passage Comprehension subtests. Internal consistency age-based reliability for both subtests range from 0.83 to 0.95. Alternate form reliability is very good for students aged eight to thirteen years, with comprehension ranging from 0.85 to 0.87 and fluency ranging from 0.91 to 0.84 [45]. The research team chose the WRMT-3 for its utility for multiple audiences. School administrators and teachers could evaluate individual student progress by percentile and grade level growth, while the test also generates the standard scores and GSV needed for rigorous analysis of group growth. Using only the fluency and comprehension subtests kept the testing time to minimum and allowed schools to evaluate reading growth of students with skills deficits for future Response-to-Intervention placement.

### Data analysis procedures

*A* priori power analysis using G*Power 3.1.9.7 was conducted to determine sufficient sample sizes for study design [46], all other data analyses were conducted using IBM SPSS version 26. As an additional guard against potential threat to internal validity *post hoc* power analyses were conducted to rule out low statistical power using G*Power 3.1.9.7 [47]. Chi-square tests for homogeneity of variance of categorical demographic variables and One-Way ANOVAs comparing pre-test scores between treatment conditions were conducted. Levene’s Tests for homogeneity of variance were evaluated.

#### Welch’s t-tests

One-Way ANOVAs indicated statistically significant differences between mean pre-test scores of the condition groups for EasyCBM comprehension, reading rate, and reading accuracy. K-S normality tests also showed non-normal distributions for EasyCBM comprehension and accuracy pre-test scores. We expected a skewed distribution for reading accuracy, as students were likely to have accuracy scores at the upper end of the scale. Due to these factors, nonparametric Welch’s *t*-tests were the most appropriate choice for examination of pre- and post-test changes of EasyCBM measures [48]. Hedge’s g effect sizes were reported for statistically significant findings: *g* ≥ 0.2 indicates a small effect, *g* ≥ 0.5 a medium effect, *g* ≥ 0.8 a large effect [49]. Pre- and post-test changes in means of EasyCBM CCSS Basic Reading raw scores, Passage Reading Fluency WCPM and reading accuracy percentages were used to examine reading comprehension and fluency skills changes within and across grade levels.

Welch’s *t*-tests of mean changes in WRMT-3 measures are reported. The homogeneity assumption was met for both WRMT-3 assessments, so two by three between-subjects factorial ANOVAs, coupled with pairwise comparisons for significant interaction effects, were used to explore research questions. Because statistical significance alone does not explain how meaningful a finding may be, effect sizes of the simple main effects were examined and reported as partial eta squared (*η*_*p*_^2^) to better understand the unique role individual variables had in the full model. Effect sizes are defined as follows: *η*_*p*_^2^ ≥ 0.01 small or no effect, *η*_*p*_^2^ ≥ 0.06 medium effect, *η*_*p*_^2^ ≥ 0.08 large effect [50].

#### Growth scale values

Age-based growth scale values (GSV) were used to examine pre- and post-test WRMT-3 fluency and comprehension changes by grade level. Whereas standard scores provide a student’s relative standing in a group and include a measure of alternate form variation, GSV are calculated on an equal interval scale with forms A and B jointly calibrated and equated. Using GSV allows for very accurate growth measurement and comparisons of change wherever they occur on the scale (i.e., across collective grade levels) [45]. The WRMT-3 also calculates grade equivalent, which quantifies months of educational progress and is readily understood by teachers and parents. Ten months of instructional growth equals one grade year level (e.g., 1.0 grade year = 10 months).

#### Factorial ANOVA

One-Way ANOVAs of all pre-test measures were performed at each grade level to determine whether intervention and typical practice condition groups were significantly different prior to intervention. The homogeneity of variance assumption was satisfied for third, fourth, and fifth grade WRMT-3 comprehension and oral reading fluency pre-tests scores. Kolmogorov-Smirnova (K-S) normality tests with Lilliefors significance correction were conducted, and test statistics and visual inspection of histograms showed WRMT-3 pre-test scores were normally distributed (Table 2). *Post hoc* two-by-three factorial ANOVA were conducted to examine change of mean (delta) and grade effects between condition groups for WRMT-3 tests.

**Table 2:** Pre-test means by intervention condition.

**Assessment**	Readable English (*N* = 441)		Typical Practice (*N* = 414)	
**EasyCBM Fall Benchmarks**	*M*	*SD*	*M*	*SD*
CCSS Reading Comprehension^a^	19.6	4.7	13.7	6.1
PRF^b^ WCPM	113.4	45.6	98.1	44.9
PRF^b^ accuracy percentage	95.5	7.1	93.9	10.4
	Readable English (*n* = 95)		Typical Practice (*n* = 98)	
**WRMT-3**	*M*	*SD*	*M*	*SD*
Passage Comprehension^c^	83.9	11.9	86.2	11.5
Oral Reading Fluency^c^	83.5	11.2	84.6	12.1

^a^Raw scores reported. ^b^PRF = Passage Reading Fluency. ^c^Standard scores reported.

## Results

Results are examined first by EasyCBM pre- and posttest changes in mean scores for all students. Students who scored at or below the 30^th^ percentile on either EasyCBM Benchmark pre-test were considered most at-risk for having significant reading skills deficits and received additional WRMT-3 Oral Reading Fluency and Passage Comprehension assessments. Mean changes in pre- and posttest GSV and grade level for that subset of students is reported following EasyCBM Benchmark changes.

### EasyCBM reading skills growth

#### Passage Reading Fluency

Students in the intervention condition outperformed students in the typical practice condition at all grade levels, demonstrating meaningful and statistically significant pre-and post-test differences on all measures of reading fluency and comprehension. The EasyCBM Passage Reading Fluency is comprised of measures of reading rate and reading accuracy. Mean changes in reading fluency from beginning to end of the school year are reported as WCPM for reading rate and as the percentage of WCPM out of total words read for reading accuracy (Table 3).

**Table 3:** EasyCBM reading benchmark test mean changes and Welch’s t-test results.

Variable	Readable English			Typical Practice			Welch’s T-Test			
	*M*	*SD*	*n*	*M*	*SD*	*n*	*t*	*df*	*p*	*g* ^a^
**Grade 3**										
PRF^b^ WCPM	28.1	21.2	136	22.2	19.4	130	2.36	263.5	.019	0.30
PRF^b^ Accuracy Percent	5.8	5.4	136	0.9	6.4	130	6.83	253.6	< .001	0.84
CCSS Comprehension	2.9	3.5	136	1.6	3.8	130	2.86	259.3	.005	0.35
**Grade 4**										
PRF^b^ WCPM	34.4	21.9	165	15.5	17.2	146	8.54	305.0	< .001	1.00
PRF^b^ Accuracy Percent	2.2	3.5	165	0.9	4.7	146	2.71	265.6	.007	0.31
CCSS Comprehension	1.6	2.8	165	0.7	4.0	146	2.16	257.3	.032	0.25
**Grade 5**										
PRF^b^ WCPM	25.3	19.7	140	14.8	23.0	138	4.08	268.6	< .001	0.50
PRF^b^ Accuracy Percent	1.4	4.4	140	0.3	1.9	138	2.72	187.4	.004	0.32
CCSS Comprehension	1.3	2.4	140	-0.4	3.3	138	4.85	249.5	< .001	0.58
**Grades 3–5 Combined**										
PRF^b^ WCPM	29.6	21.333	441	17.4	20.2	414	8.60	852.9	< .001	0.59
PRF^b^ Accuracy Percent	3.1	4.8	441	0.7	4.7	414	7.32	852.4	< .001	0.50
CCSS Comprehension	1.9	3.0	441	0.6	3.8	414	5.39	784.3	< .001	0.37

^a^Hedge’s *g* effect sizes in context: *g* ≥ 0.2 indicates small/no effect, *g* ≥ 0.5 indicates medium effect, *g* ≥ 0.8 indicates large effect.

^b^PRF = Passage Reading Fluency.

**Fig 2:**
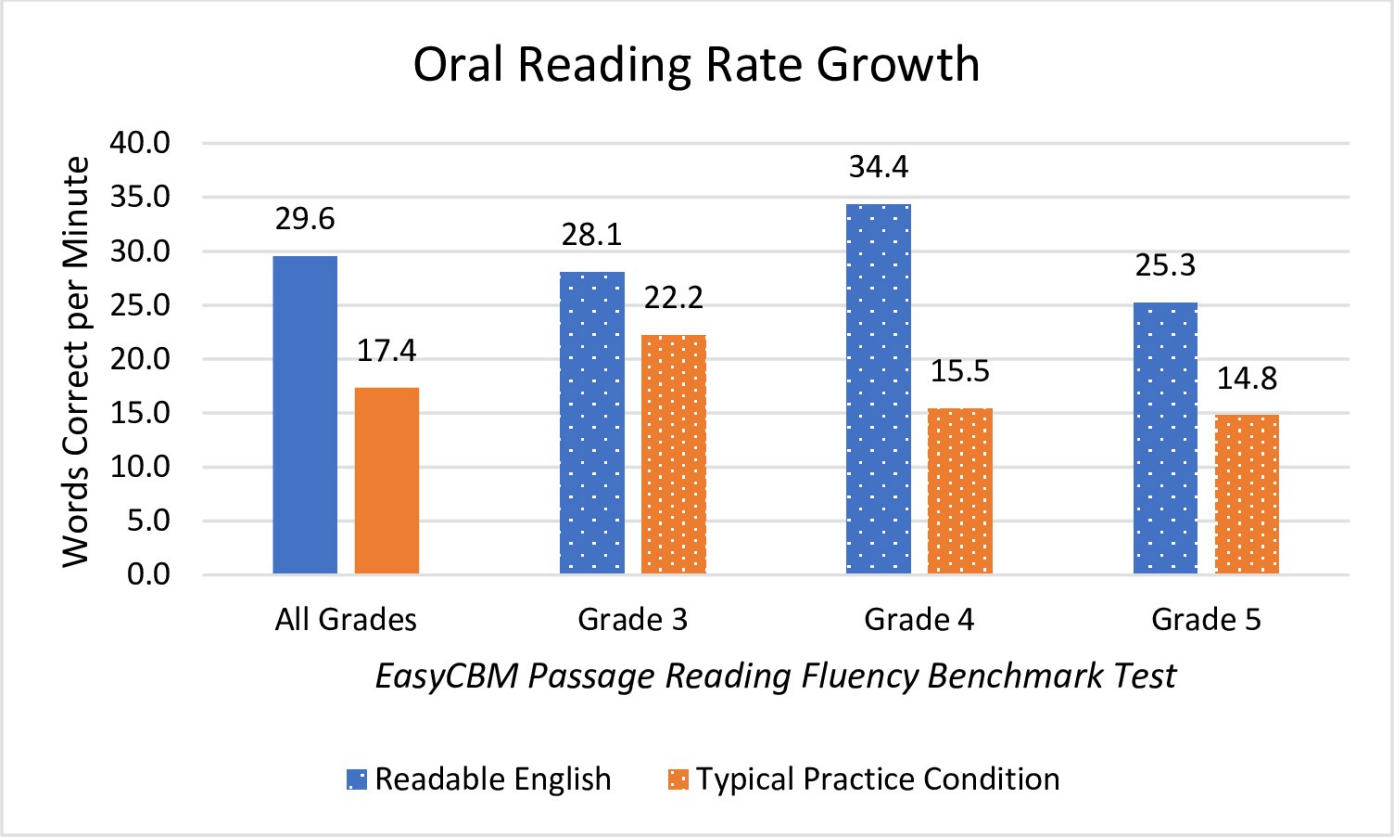
Mean WCPM changes in EasyCBM Passage Reading Fluency. Graph shows net mean changes reported for all grades combined and individually by grade level.

Students in the intervention practice significantly outperformed students in the typical practice condition at each individual grade level. There was a moderate effect size for WCPM reading rate for the combined grade levels with the biggest disparity between conditions seen in fourth grade where students in the Readable English intervention condition averaged about 19 more WCPM out of mean 145 WCPM than did peers in the typical practice condition (see Fig 2 for reading rate).

**Fig 3:**
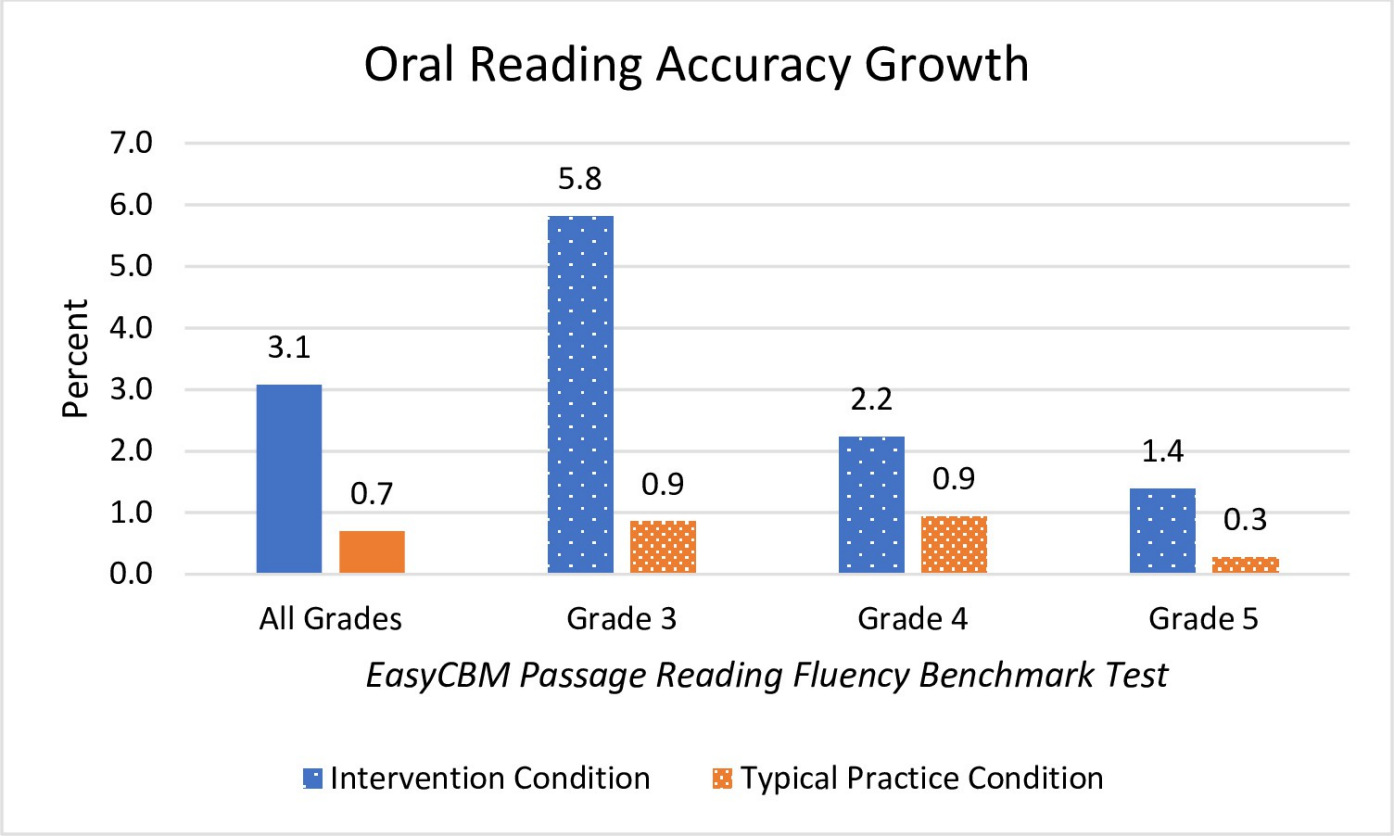
Mean changes in EasyCBM Passage Reading Fluency accuracy. Reading accuracy is measured as the percentage of WCPM out of total words read. Graph shows net mean changes reported for all grades combined and individually by grade level.

The improved reading accuracy of all grades combined demonstrated a moderate effect size with students in the Readable English condition reading 3.1% more accurately at the end of the school year than students in the typical practice condition who improved only 0.7% (Fig 3). Third grade showed the most improvement in reading accuracy with the intervention group reading about 5.0% more accurately than students in the typical practice condition. Reading accuracy and reading rate are vital to good comprehension. Misreading several words out of every hundred words can significantly change the meaning of the text. Two main factors of good reading include quick and accurate oral reading. Other important, but unmeasured factors in this study include prosody, word recognition, and vocabulary knowledge. While all grades, both individually and when grouped, showed superior reading skills gains for the Readable English condition, no single grade outperformed another.

#### CCSS Basic Reading Comprehension

Students in the Readable English intervention surpassed the reading comprehension of students in the typical practice condition with a moderate effect size. *Post hoc* linear regression of combined grades showed that improvements in EasyCBM Passage Fluency reading rate and accuracy accounted for 14% of the improvement in CCSS Reading Comprehension raw scores (*F*(3,851 = 47.01), *p*<0.001, adjusted *R*^2^ = 0.14). Combining the three grades for an overarching analysis showed that reading comprehension was meaningfully improved for the Readable English intervention group with a small effect size and large observed power.

**Fig 4:**
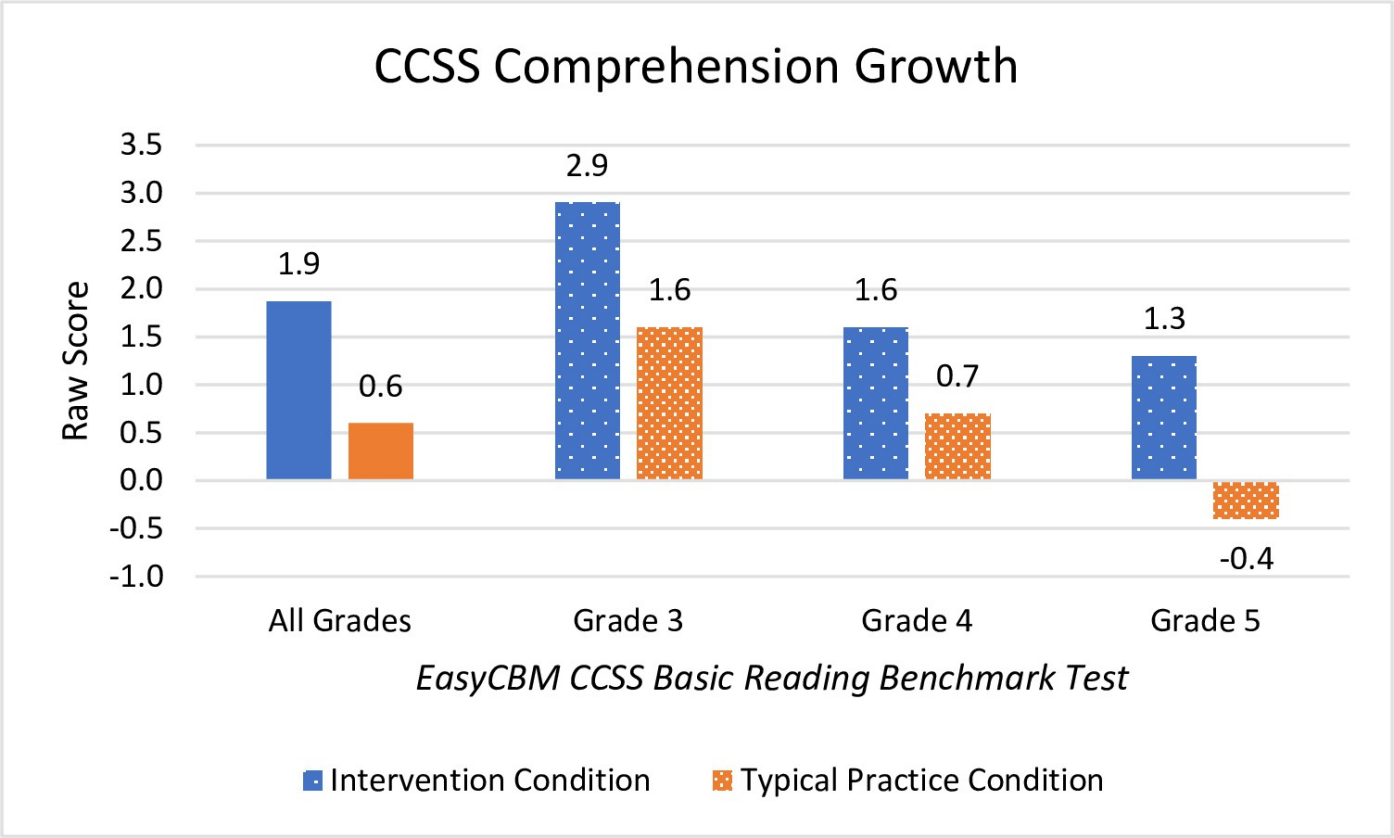
Mean raw score changes in EasyCBM CCSS Basic Reading Comprehension. Graph shows net mean changes reported for all grades combined and individually by grade level. Negative score means indicate learning loss for students who did not grow academically compared to same-grade peers.

Examination of reading comprehension growth by grade level shows the largest difference in mean change between groups lies with fifth grade (Δ_*M*_ = 1.7) where there was a moderate effect size (Hedge’s g = 0.6) compared to students in the typical practice condition who showed net learning loss on this measure (Δ_*M*_ = -0.4). Students demonstrating learning loss have not necessarily forgotten prior learning, but instead have failed to grow at the same rate as their peers. Fig 4 shows the reading comprehension growth of raw scores for both condition groups.

### WRMT-3 reading skills growth

#### Oral Reading Fluency

**Fig 5:**
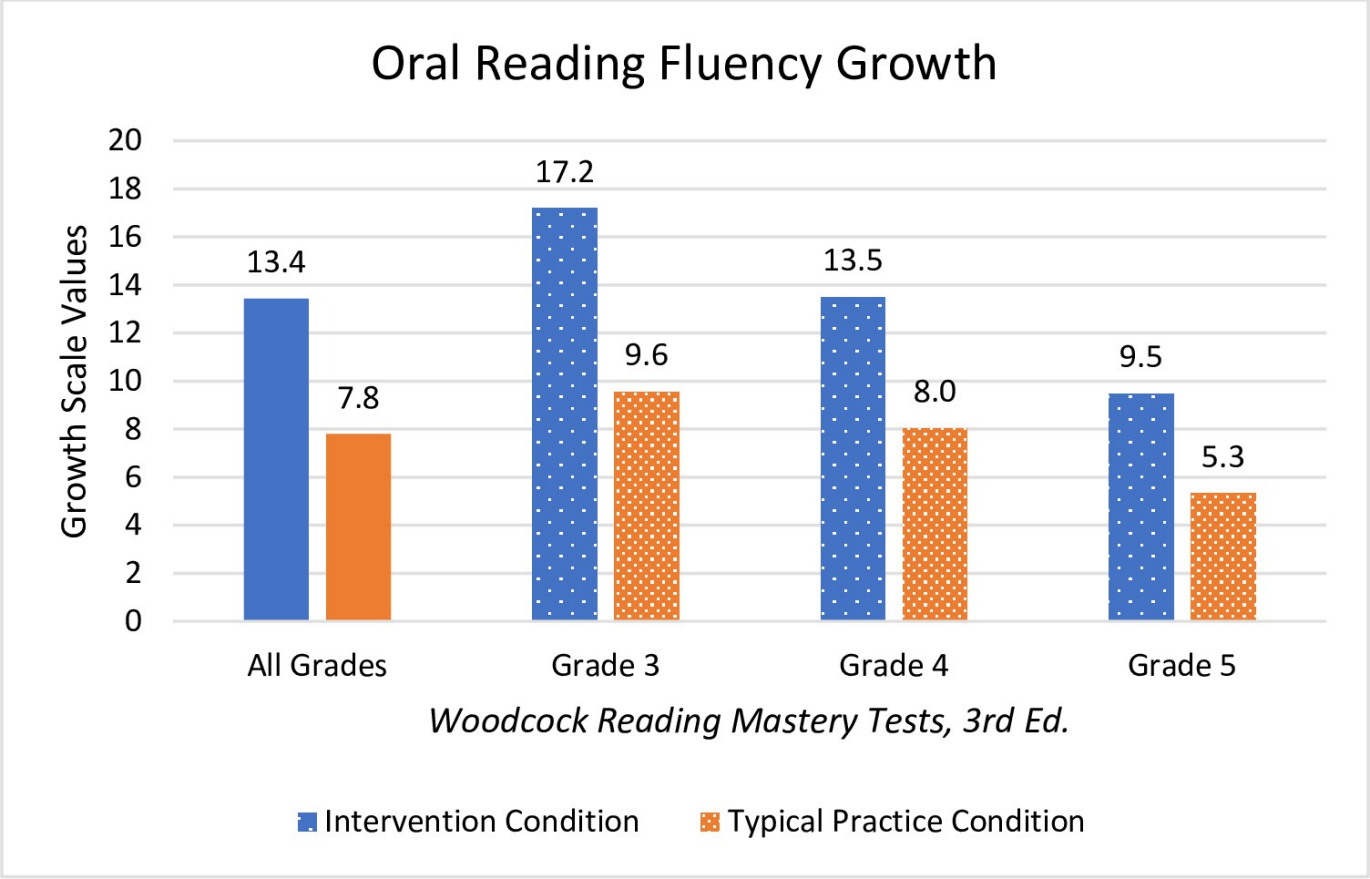
Mean GSV changes in WRMT-3 Oral Reading Fluency. Graph shows net mean changes in GSV reported for all grades combined and individually by grade level.

Students in the Readable English condition meaningfully and significantly outperformed students in the typical practice condition on Oral Reading Fluency. Table 4 shows pre-test and posttest means, change in means, *t* test results, and effect sizes, while Fig 5 displays WRMT-3 Oral Reading Fluency outcomes graphically. The overall result is that students in the Readable English condition experienced meaningful gains in reading fluency. Multilevel ANOVA showed there was not a significant effect of grade level on Oral Reading Fluency scores (*F*(2, 190) = 0.66; *p* = 0.519), meaning all three grades experienced similar growth in oral reading fluency. That is an unusual finding because oral reading fluency growth typically starts to slow as students engage with increasingly complex text. Visual inspection of scatter plots shows that participants in both condition groups had plenty of room for fluency growth (i.e., no ceiling effects were seen), and students in the Readable English condition experienced remarkable fluency growth at all grade levels (Table 4).

**Table 4:** WRMT-3 Oral Reading Fluency and Passage Comprehension mean GSV changes and t-test results.

Variable	Readable English			Typical Practice			Welch’s T Test			
	*M*	*SD*	*n*	*M*	*SD*	*n*	*t*	*df*	*p*	*g* ^a^
**Grade 3**										
ORF^b^	17.2	7.6	32	9.6	10.5	33	3.38	58.33	< .001	0.83
PC^c^	9.4	11.1	32	7.2	14.7	33	0.70	59.56	.492	0.17
**Grade 4**										
ORF^b^	13.5	11.4	32	8.0	6.6	38	2.40	47.63	.020	0.60
PC^c^	12.4	9.0	32	6.6	11.2	38	2.44	67.89	.018	0.57
**Grade 5**										
ORF^b^	9.5	6.9	31	5.3	7.2	27	2.24	54.33	.029	0.58
PC^c^	11.2	8.9	31	-2.1	8.1	27	5.95	55.93	< .001	1.53
**Grades 3–5 Combined**										
ORF^b^	13.4	9.3	95	7.8	8.3	98	4.43	187.0	< .001	0.64
PC^c^	11.0	9.7	95	4.4	12.3	98	4.15	183.5	< .001	0.59

^a^Hedge’s *g* effect sizes in context: *g* ≥ 0.2 indicates small/no effect, *g* ≥ 0.5 indicates medium effect, *g* ≥ 0.8 indicates large effect. ^b^ORF = Oral Reading Fluency. ^c^PC = Passage Comprehension.

Significant and large main effects for WRMT-3 Oral Reading Fluency were found *F*(5,187 = 7.49, *p* = < .001, partial η^2^ = .167. There were moderately strong main effects of grade *F*(2,187) = 7.44, *p* = < .001, partial η^2^ = .074 and of typical practice condition *F*(1,187) = 21.67, *p* = < .001, partial η^2^ = .104. *Post hoc* pairwise comparisons showed a significant interaction of Oral Reading Fluency and grade level, but only between grades three and five. This follows expectations for typical fluency growth, as younger grades show steeper reading rate growth with growth of WCPM gradually leveling off around grade six. Typically developing readers express larger WCPM at grade three and smaller WCPM gains in grades five and up as text complexity increases and instructional focus shifts from learning-to-read-fluently to reading-fluently-for-comprehension. The gains in reading rate and accuracy experienced by students in the Readable English condition are considerable and will give exponential word reading volume dividends allowing students to read text faster and more accurately going forward.

#### Passage Comprehension

**Fig 6:**
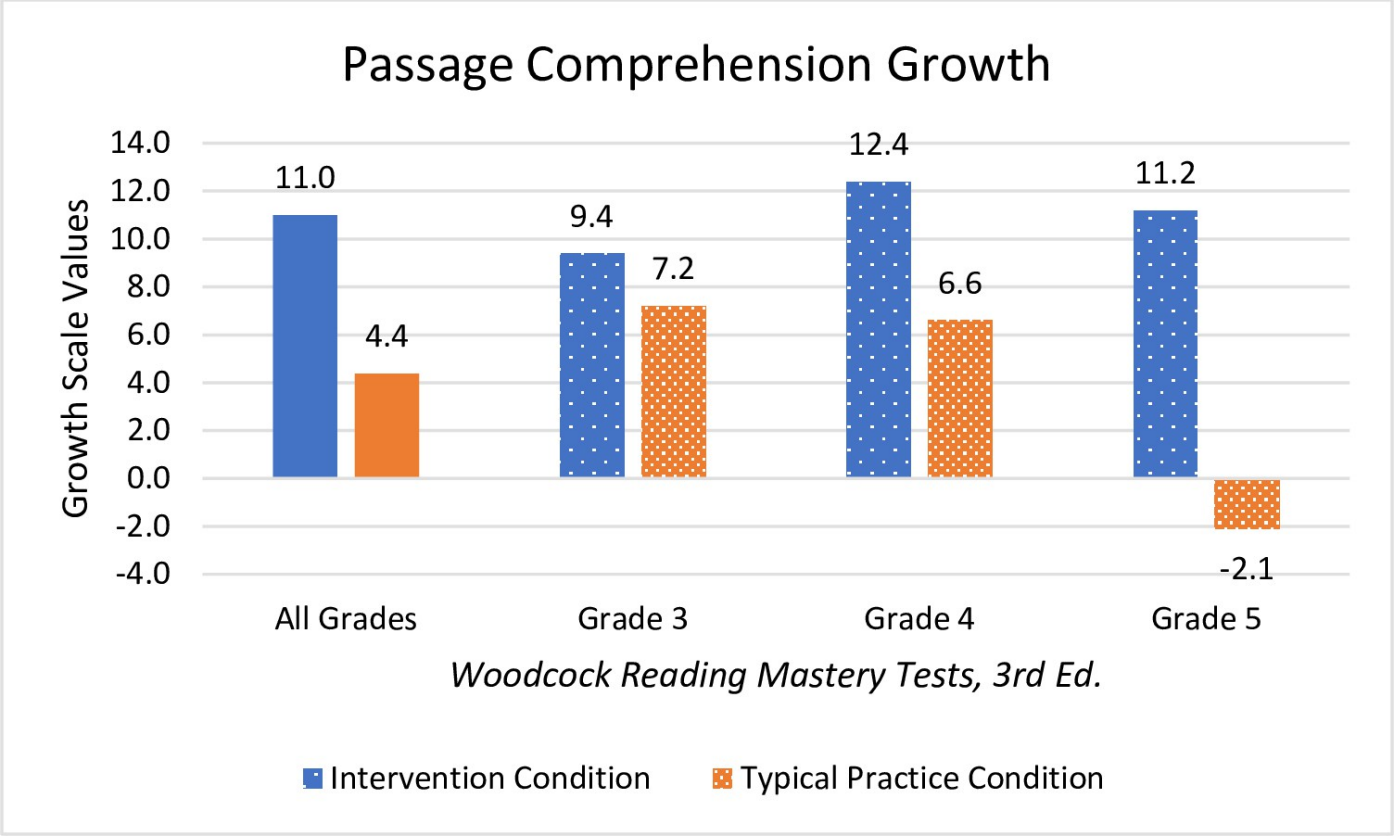
Mean GSV changes in WRMT-3 Passage Reading Comprehension. Graph shows net mean changes in GSV reported for all grades combined and individually by grade level. Negative score means indicate learning loss for students who did not grow academically compared to same-grade peers.

Significant and large main effects for WRMT-3 Passage Comprehension were found *F*(5,187 = 6.55, *p* = < .001, partial η^2^ = .149, meaning participants in the Readable English condition had meaningful comprehension growth relative to students in the typical practice condition (see Fig 6). There was also a large main effect of treatment condition *F*(1,187) = 20.69, *p* = < .001, partial η^2^ = .100 and a small effect of grade level *F*(1,187) = 3.53, *p* = .031, partial η^2^ = .36. The interaction of treatment condition and grade level was significant with a moderate effect size *F*(2, 187) = 4.09, *p* = .018, partial η^2^ = .042. Pairwise comparisons by grade show that the only significant interaction was the difference between grades four and five *t* = 2.58, *p* = .011, negating the probability of a true interaction between treatment condition and grade level. Students in all three grade levels in the Readable English condition made substantial gains in reading comprehension with growth increasing incrementally from third through fifth grades, however the differences between reading comprehension growth in third grade was not statistically significant. Overall analyses for the combined grades showed reading comprehension gains for students in the Readable English condition were statistically significant, large, and meaningfully improved students’ reading abilities.

**Fig 7:**
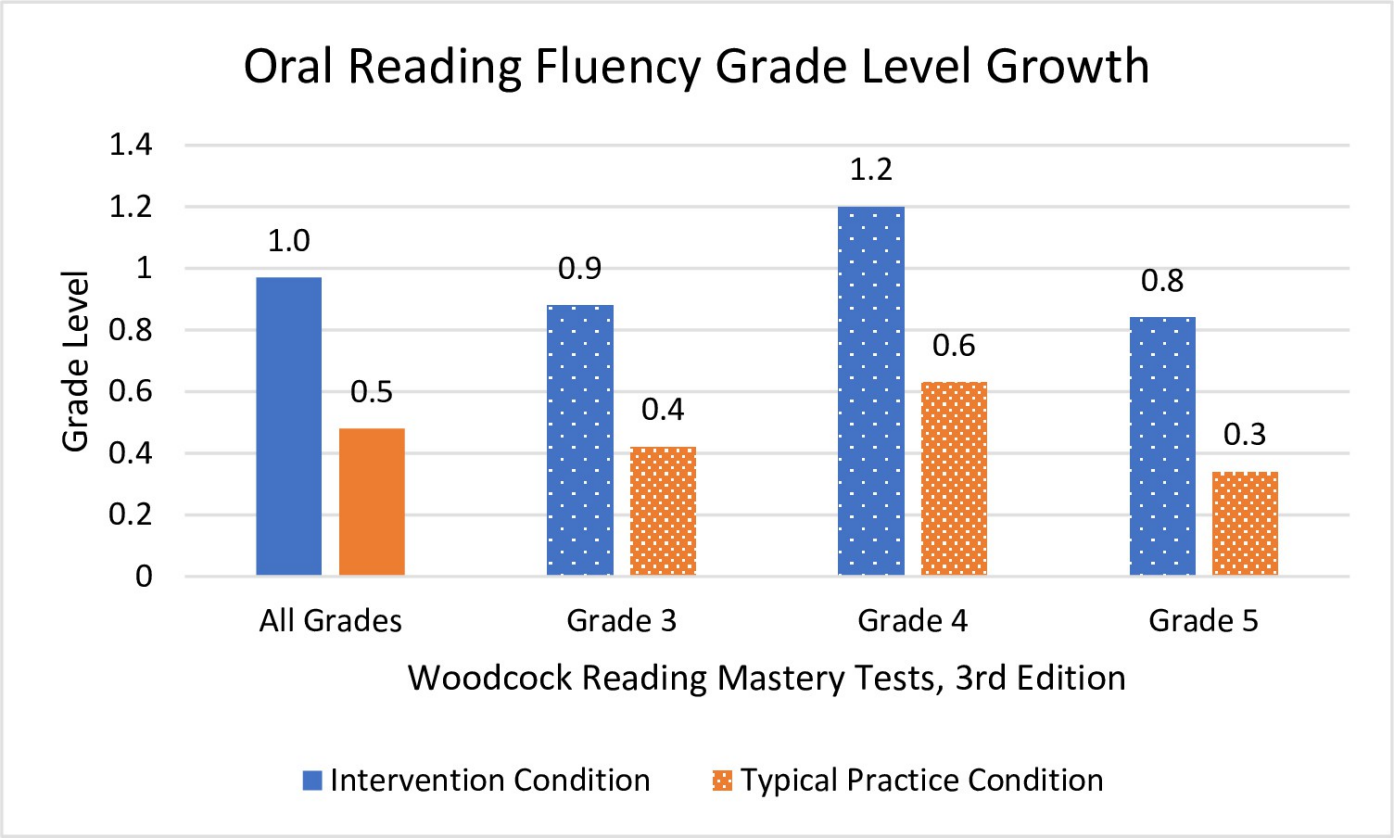
Mean grade level changes in WRMT-3 Oral Reading Fluency. Each tenth of a grade level equals one month of growth.

**Fig 8:**
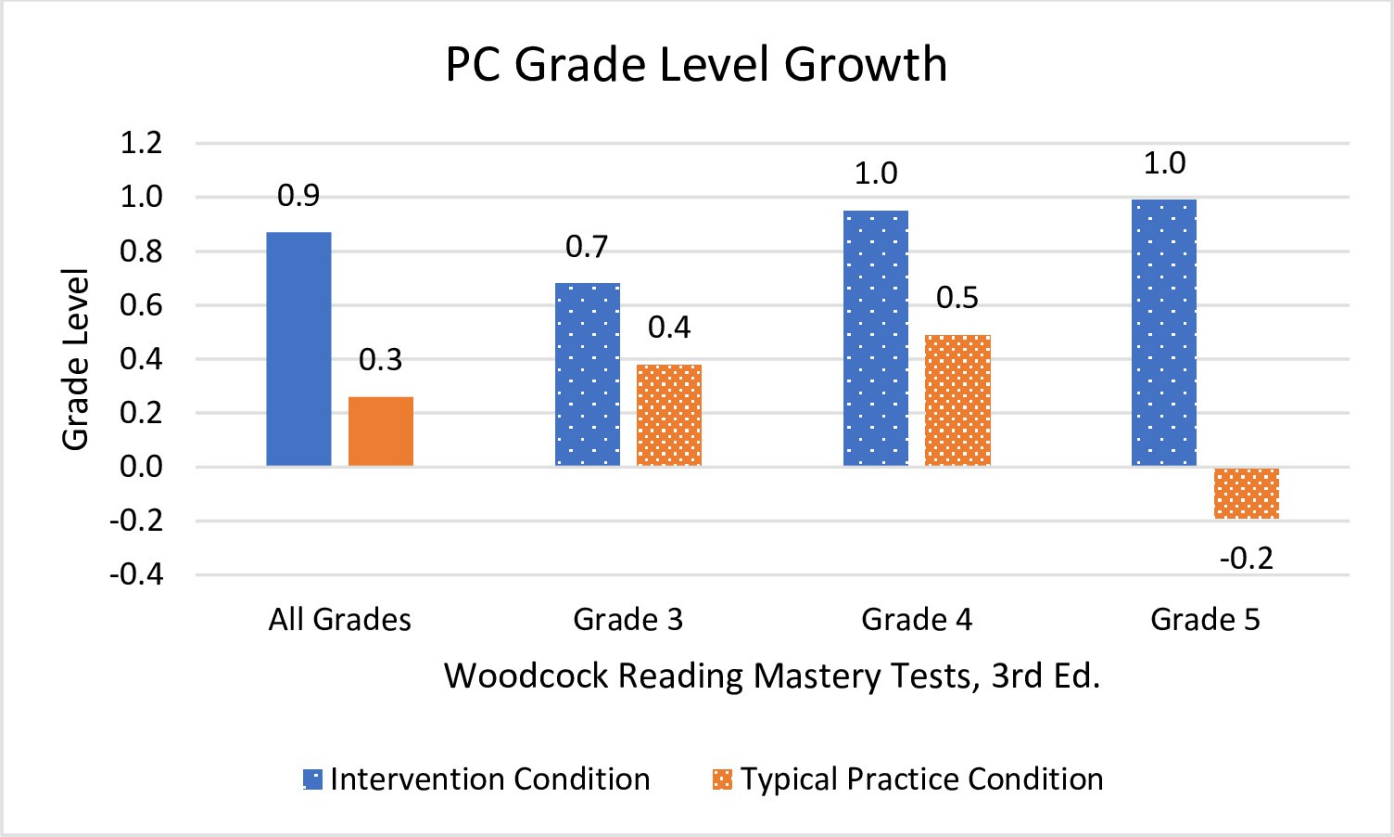
Mean grade level changes in WRMT-3 Passage Reading Comprehension. Each tenth of a grade level equals one month of growth. Negative score means indicate learning loss for students who did not grow academically compared to same-grade peers.

The prior results are analyses of raw scores, GSV, and percentages designed to root out the efficacy of the Readable English intervention. Examination of effect sizes, probability, *F* and *t* values, and statistical power indicate these findings were statistically significant, and they also represented meaningful educational gains for students. School leaders, however, want to know if Readable English is powerful enough to help students who are reading below grade level close reading gaps. Teachers and parents want to know “How many grade levels did my students grow?” Examination of the mean change in grade equivalents provided by WRMT-3 give an idea of grade level growth for students in both condition groups. Table 5 shows the mean changes in grade equivalents, while Figs 7 and 8 graph those differences visually. Observed power was adequate for each test, ranging from 0.60 to 0.99.

**Table 5:** WRMT-3 Oral Reading Fluency and Passage Comprehension mean grade level changes and Welch’s t-test results.

Variable	Readable English			Typical Practice			Welch’s T Test			
	*M*	*SD*	*n*	*M*	*SD*	*n*	*t*	*df*	*p*	*g* ^a^
**Grade 3**										
ORF^b^	0.9	0.7	32	0.4	0.5	33	3.04	56.7	.004	0.76
PC^c^	0.7	0.7	32	0.4	1.1	33	1.27	54.9	.209	0.32
**Grade 4**										
ORF^b^	1.2	1.1	32	0.6	0.7	38	2.53	48.3	.015	0.63
PC^c^	1.0	1.0	32	0.5	0.8	38	2.11	60.4	.039	0.51
**Grade 5**										
ORF^b^	0.8	1.00	31	0.3	0.6	27	2.31	51.2	.025	0.58
PC^c^	1.0	0.8	31	-0.2	0.7	27	5.77	54.7	< .001	1.49
**Grades 3–5 Combined**										
ORF^b^	1.0	1.0	95	0.5	0.6	98	4.22	160.3	< .001	0.61
PC^c^	0.9	0.9	95	0.3	0.9	98	4.69	184.8	< .001	0.68

^a^Hedge’s g effect sizes in context: *g* ≥ 0.2 indicates small/no effect, *g* ≥ 0.5 indicates medium effect, *g* ≥ 0.8 indicates large effect. ^b^ORF = Oral Reading Fluency. ^c^PC = Passage Comprehension. One year’s growth on the is one grade level (1.0), which is equal to ten months.

The big picture view of grade level reading growth is that when mean changes in grade equivalents for all grades are combined, students who received Readable English instruction greatly outperformed students in the typical practice condition. Combining the grade levels increased the sample size for analysis so that observed power was strong. Mean changes in grade equivalents for individual grade levels are reported, but the sample sizes are so small that observed power for those tests is weak, and grade three Passage Comprehension is not statistically significant. Prior data all indicate the intervention group realized significant and meaningful reading skills gains compared to the typical practice group.

This summary analysis of mean changes in grade equivalents serves only to put those gains into a practical perspective for school leaders. Analysis of the WRMT-3 Oral Reading Fluency subtest showed that the combined grades grew approximately ten months in the intervention condition and five months in the typical practice condition. Expected annual growth is ten months, so students receiving Readable English averaged one year’s oral reading fluency growth, while students in the typical practice condition did not. Students in the Readable English condition gained approximately nine months of reading comprehension growth compared to three months gained by students in the typical practice condition. In a year typified by learning loss, improving reading skills by ten months *is* closing reading gaps.

## Discussion

Children who are not proficient readers cannot fully participate in and access all available educational opportunities, and 67–69% of American students at fourth and eighth grade assessment points are not able to read proficiently enough to fully engage with grade level curriculum [1]. Achieving social equity will not happen until educational equity is realized; and equity in education begins with making certain all students are proficient readers. Growing students into proficient readers requires sustainable, research proven reading programs designed to support the wide variety of skills processes involved in reading.

Though the mechanics of reading are complex [10], reading research provides abundant evidence of the skills involved and where things can go awry in the process of learning to read [7, 28, 29]. Reading programs that provide individualized, multiple component skills instruction are particularly effective for students beyond second grade [2]. This research study confirms and extends prior research by Fogarty et al. (2014) [36], Lovett et al. (2021 and 2000) [8, 23], Scammacca et al. (2016) [4], and Solis et al. (2014) [51] of the particular effectiveness of multiple component reading programs for adolescent readers.

Just as reading fluency spirals into increasing vocabulary knowledge and reading comprehension, reading instruction must be cyclical to address ongoing and evolving student reading needs. To address these needs teachers should be provided with reading programs proven to meet student needs that vary widely across multiple grade levels. Effective reading programs must offer a continuous path forward to support students at their current reading ability level as they read core curriculum and become increasingly skilled readers. Ongoing reading skills instruction that is embedded in the content area reading material helps all learners improve their reading and keeps many students from needing pull-out remedial reading instruction. In addition to being very effective, Readable English is educationally sustainable for our increasingly overburdened educators.

Despite pandemic instructional interruptions, students in grades three, four, and five in the Readable English intervention conditions experienced meaningful improvement on all measures of oral reading fluency, reading rate, reading accuracy, and passage reading comprehension compared to students in the typical practice condition who also received instruction using a research proven multiple component reading program. Readable English has high utility for elementary teachers who need to scaffold reading instruction for learners with wide ranging learning needs. This is particularly important in the context of widespread learning loss due to the COVID-19 pandemic. Estimates for remediating just 30% of pandemic learning loss range from $800 to $3,800 per student [52].

Students who received Readable English instruction flourished from the combined effect of asset-based, multiple component reading skills instruction, averaging one year’s growth in reading fluency and nine months’ growth in reading comprehension with less than 60 hours of instruction. These findings add further evidence to prior research in the areas of asset-based instruction and multiple component reading instruction. More important though, is the evidence that Readable English can help students learn to read. On EasyCBM measures, overall reading accuracy improved more than 3% for students in the Readable English condition, which is about nine more words read correctly per double spaced page. Post-intervention, students who received Readable English instruction also read 22 WCPM (out of mean 142 WCPM) more than students in the typical practice condition.

Similarly, the WRMT-3 assessments show that students in the Readable English condition grew 13.4 GSV on Oral Reading Fluency measures compared to students in the control group who averaged 7.8 GSV, representing a medium effect size for the combined grades. The extensive fluency growth of students in the intervention condition boosted Passage Comprehension to an average growth of 11.0 GSV (medium effect size for all grades), while the anemic growth of fluency growth by students in the control condition translated to an average 4.4 GSV growth in their comprehension. The robust growth students in the intervention condition experienced during this study shows that Readable English can close reading skills gaps for students during the regular school day for a fraction of those eye-watering learning loss remediation cost estimates.

Faster and more accurate reading fluency was accompanied by grade equivalent gains in reading comprehension for Readable English students. As with prior studies showing that fluency instruction benefits students’ reading comprehension even when they have average reading fluency [8, 22], students in this study’s intervention condition also experienced meaningful comprehension gains. By supporting foundational reading skills development, Readable English instruction helped students to develop strong reading comprehension skills. The intervention’s individualized reading supports enabled students to access grade level content while simultaneously offering enrichment opportunities for skilled readers to successfully navigate more difficult text.

These research findings add evidence to Ladson-Billings’ (2006) [9] theory that using an asset-based approach to reading instruction that builds on the interests and strengths of students greatly benefits young readers. Students who use Readable English have increased autonomy and ownership of their own reading by opting to convert difficult text or material they find personally relevant and interesting into the Readable English mark-up. That is the very nature of asset-based reading instruction with the added bonus of student autonomy and control of their own learning. This process individually scaffolds students so they can continually read “up” to the level of their strengths rather than reading “down” at the level of their skills deficits.

### Study strengths, limitations, and future research

Robust sample sizes, large and comparable treatment and control conditions, and a moderate length of instructional time are all study strengths. The use of both normed grade level benchmarks and standardized reading assessments allowed the close comparisons between groups to discover the extent of reading skills improvements. The ability to compare grade level reading fluency and comprehension growth of students who need to close significant reading gaps is a vital element; while use of GSV added statistical strength and precision needed to compare groups across grade levels.

Several factors should be considered before attempting to generalize these study findings. First, study participants were not demographically diverse: all students lived in rural areas and most students were white. Future research should include more diverse populations including multilingual learners to better ascertain the program’s generalizability. Additional measures should be considered in future studies. Teacher and student perceptions of the intervention were not measured That qualitative data would help provide insight into teacher and student attitudes about aspects of Readable English. Motivation is often an undervalued factor in learning, and future studies might benefit from a survey of student reading interest and motivation. One of the perceived strengths of Readable English is that students and teachers can use the conversion tool and choose the text they want to read. Providing students and teachers with choice and voice about what text to read should increase the fun factor for students and the utility of the program for teachers, but without that qualitative data this is pure speculation.

Technical improvements in the Readable English usage database could enable future researchers to compare aspects of intervention usage (i.e., number of words read, number of text conversions, time on site, number of passages read) to skills improvements. The company database was under revision during this research study, so I was unable to determine which types of online activities might be positively correlated with measures of reading fluency or comprehension. There should be a strong correlation between word reading volume and increased oral reading fluency, passage comprehension, and vocabulary skills growth. Future studies should consider including a robust measure of vocabulary as a mediator of decoding and comprehension [28].

As with other current long-term studies, the biggest limitations stem from the educational disruptions. Participant attrition, though not significantly different between treatment conditions, diminished group sample sizes. Teacher fatigue probably impacted both treatment conditions, and it remains an unquantified factor; and lost instructional time significantly reduced the planned intervention time. Due to the loss of instructional time, students and teachers did not use the Readable English conversion tool as initially planned. While students in the intervention group showed excellent reading skills growth during the forty-five to sixty hours of received instruction, we cannot know how additional intervention instruction would change the slope of the reading trajectory. Would we see increasing benefits over time, or would learning plateau?

## Conclusion

Readable English is an excellent tool to accelerate reading growth. The program is effective across multiple grade levels because it remediates multiple linguistics skills deficits while providing individualized scaffolding for students in core curriculum. Because the program is easy to use, is scripted, and comes with complete lesson plans, training supports, and individual and small group instructional activities, teachers can implement the program with fidelity. All program content is available online, so teachers do not have to store books or carry heavy books or manuals home for weekend lesson planning. The student Progress, Data, and Reports tab makes it easy to track individual and class progress. Students willingly engage with and enjoy using Readable English, elements that are critical for successful learning. Being highly effective, easy for teachers, liked by students, and affordable for districts and homeschool parents alike makes Readable English a viable solution to the national reading crisis.

The children we fail to teach to read proficiently are statistically likely to grow into adults with low literacy skills that face a myriad of social, economic, health, and emotional hardships and limitations [3, 14, 15, 25, 53]. The fallout from not being able to read well is an education debt that lasts a lifetime and impacts the whole family [9]. There is a well-documented reading crisis in the United States that makes finding and using research proven reading programs critically important. This study shows that Readable English is highly effective for third, fourth, and fifth grade students, offers a solution to our literacy quagmire, and adds to the body of research into multiple component reading programs.

The gains in reading rate and accuracy experienced by students in the Readable English condition are considerable and will give exponential word reading volume dividends to students able to read text faster and more accurately going forward. Ongoing reading skills instruction that is embedded in the content area reading material helps all learners improve their reading and keeps many students from needing pull-out remedial reading instruction. In addition to being very effective, Readable English is educationally sustainable for our increasingly overburdened educators.

## Supporting information

S1 File Overview video of Readable English. Readable English© (2023). Reprinted with permission.
(MP4)

S2 File Readable English eReader functionality video. Readable English© (2023). Reprinted with permission.
(MP4)
